# Machine learning-based integration develops an immunogenic cell death-derived lncRNA signature for predicting prognosis and immunotherapy response in lung adenocarcinoma

**DOI:** 10.1038/s41598-024-62569-z

**Published:** 2024-05-22

**Authors:** Jiazheng Sun, Hehua Guo, Siyu Zhang, Yalan Nie, Sirui Zhou, Yulan Zeng, Yalu Sun

**Affiliations:** 1 Department of Respiratory Medicine, Liyuan Hospital, Tongji Medical College, Huazhong University of Science and Technology, Wuhan, China; 2 Department of Rehabilitation Medicine, Affiliated Hospital of Jining Medical University, Jining, China; 3 Department of Respiratory Medicine, The First People’s Hospital of Jiangxia District, Wuhan, China

**Keywords:** Lung adenocarcinoma, Immunogenic cell death, LncRNA, Prognostic signature, Immunotherapy, Cancer, Computational biology and bioinformatics, Diseases

## Abstract

Accumulating evidence demonstrates that lncRNAs are involved in the regulation of the immune microenvironment and early tumor development. Immunogenic cell death occurs mainly through the release or increase of tumor-associated antigen and tumor-specific antigen, exposing “danger signals” to stimulate the body’s immune response. Given the recent development of immunotherapy in lung adenocarcinoma, we explored the role of tumor immunogenic cell death-related lncRNAs in lung adenocarcinoma for prognosis and immunotherapy benefit, which has never been uncovered yet. Based on the lung adenocarcinoma cohorts from the TCGA database and GEO database, the study developed the immunogenic cell death index signature by several machine learning algorithms and then validated the signature for prognosis and immunotherapy benefit of lung adenocarcinoma patients, which had a more stable performance compared with published signatures in predicting the prognosis, and demonstrated predictive value for benefiting from immunotherapy in multiple cohorts of multiple cancers, and also guided the utilization of chemotherapy drugs.

## Introduction

According to worldwide data from 2018, lung cancer is one of the most common kinds of cancers and is more common in men than in women, which is the second cause of mortality for women behind breast cancer, making it the third most common malignancy overall^1^. The most prevalent form of lung cancer is lung adenocarcinoma (LUAD), which has displayed an increasing incidence in recent years^2^. With recent progress in the study of the tumor microenvironment and the development of tumor immunotherapy, especially as the treatment of immune checkpoint inhibitors has become the standard of treatment for patients with advanced non-small cell lung cancer, it is imminent to the search for more precise signature to assess the value of immunotherapy.

Immunogenic cell death (ICD) is a cell death modality that stimulates an immune response against dead-cell antigens^3^, which prompts dendritic cells (DC) to mature and present antigens to Cytotoxic T Lymphocytes (CTL), thereby activating CTLs to clear neighboring cells^4^. In addition, ICD is deemed to overcome the drug resistance of tumors by mediating the immune response and enhancing the anti-tumor immunity of CTL to optimize the therapeutic effect^5–7^.

Transcripts with a length greater than 200 nucleotides are referred to as long non-coding RNAs (lncRNAs)^8^, which participate in several biological processes^9–11^. Accumulating pieces of evidence demonstrate that lncRNAs exert an enormous function on several primary regulatory processes, such as genomic imprinting, chromatin modification, and transcriptional activation in recent years^12^. In addition, Studies have proved that lncRNAs manipulate early tumor development through different signaling pathways, for example, lncRNA MEG3 can suppress tumor progression by promoting the expression of P53^13^. lncRNA PCGEM1 may instruct c-Myc to bind to the promoters of genes associated with metabolism to increase their expression, which in turn contributes to the glucose uptake and anaerobic glycolysis in tumor cells and manipulates the metabolic reprogramming of tumors^14^.

**Figure 1:**
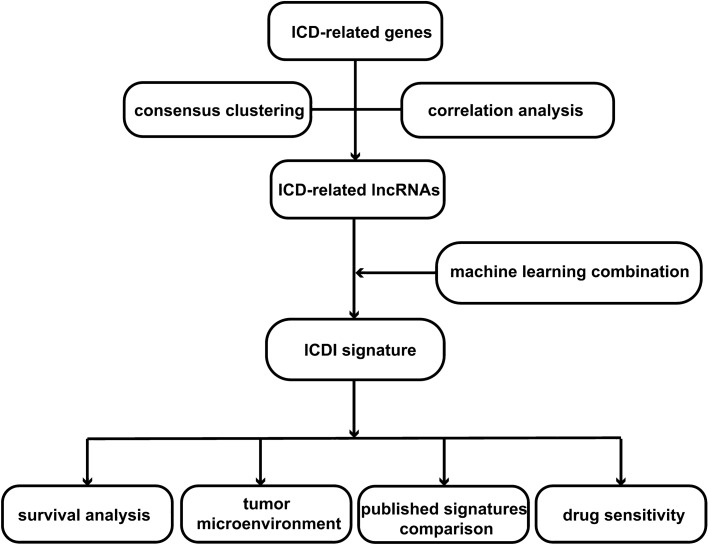
The study’s flowchart diagram.

The procedure of this study is shown in Fig. 1. We employed Pearson correlation analysis, consensus cluster analysis, differential expression analysis, and univariate regression analysis to identify ICD-related lncRNAs. The most effective ICDI signature was subsequently established by consolidating the results obtained from 101 machine-learning algorithms. The reliability of ICDI signatures is assessed by combining TCGA and several GEO data sets and using AUC and C-index measures. The superiority of this ICDI signature is evaluated by comparing it with 102 published ICDI signatures. Hence, we conducted a study to examine the correlation between ICDI attributes and immune-related attributes, as well as the sensitivity of chemotherapeutic drugs. The aim was to evaluate the potential influence of ICDI on guiding both immunotherapy and chemotherapy. Ultimately, qrt-PCR was employed to initially validate the expression of various ICDI-associated long non-coding RNAs in lung cancer cells.

## Materials and methods

### Processing and collection of data

The training cohort consisted of RNA-Seq data obtained from 493 LUAD patients, which were retrieved from the TCGA GDC site (https://www.tcga.org). As validation cohorts, five datasets were downloaded from the GPL570 platform in the GEO database (http://www.ncbi.nlm.nih.gov/geo/). These data sets are GSE29013^15^, GSE30219^16^, GSE31210^17^, GSE3141^18^, and GSE50081^19^. The definitive clinical features of datasets were elaborated in Supplementary Table 1. The patients in the training cohort and validation cohorts were selected based on the following criteria: 1. Individuals were diagnosed with lung adenocarcinoma. 2. The patient possesses explicit survival data. 3. The patient’s survival time is nonzero. The “hgu133plus2” software is utilized to annotate GEO data sets. Utilize the “IOBR” package to transform FPKM values and Count values into TPM values.

The somatic mutation data from the TCGA-LUAD cohort was obtained in MAF format from UCSC Xena (https://xena.ucsc.edu/). The R software package “maftools” was utilized to investigate the nature and occurrence rate of DNA mutations.

The treatment information and RNA-seq data of the immunotherapy cohort were obtained from the GEO dataset and published literature, specifically Melanoma-GSE78220, STAD-PRJEB25780, and GBM-PRJNA482620.

### Integration of machine learning algorithms

To develop a signature with high accuracy and stability performance, we integrated 10 machine learning algorithms including random survival forest (RSF)^20^, elastic network (Enet)^21^, Lasso, Ridge, Stepwise Cox^21^, CoxBoost^22^, partial least squares regression for Cox (plsRcox)^23^, supervised principal components (SuperPC)^24^, generalized boosted regression modeling (GBM)^25^, and survival support vector machine (survival-SVM)^26^. A few algorithms possessed the ability of feature selection, such as Lasso, Stepwise Cox, CoxBoost, and RSF. Thus, we combined these algorithms to generate a consensus model. In total, 101 algorithm combinations were conducted to fit prediction models via tenfold cross-validation.

### Functional enrichment analysis

The R package “clusterProfiler” was used to conduct gene ontology (GO) enrichment analysis and Kyoto Encyclopedia of Genes and Genomes (KEGG) enrichment analysis for ICD-related genes. the results of adjusted Pvalue < 0.05 were considered statistically significant.

### Validation and modeling of ICDI signature

Consensus clustering was employed in the TCGA-LUAD cohort to detect clusters based on gene expression profiles associated with ICD. The “ConsensusClusterPlus” package does this procedure. The best cluster number was determined using a mix of consensus scoring matrix, CDF curve, PAC score, and Nbclust. The “limma” software was used to identify lncRNAs that were expressed differently in various clusters. These lncRNAs were then compared with the lncRNAs obtained by Pearson correlation analysis (|R|> 0.3, P < 0.05) to identify ICD-related lncRNAs. A total of 101 machine-learning algorithm combinations were generated based on ICD-related lncRNAs. The C-index was derived from the coxph result of the “survival” package and represents the performance of the machine learning algorithm and clinical features. The ideal ICDI signature was determined by selecting the highest value of the C-index. The samples were categorized into two groups based on the median ICDI score. Subsequently, a Kaplan–Meier analysis was conducted. The “timeROC” software package was utilized to build receiver operating characteristic (ROC) curves that fluctuate over time. These curves were used to assess the accuracy of prognostic characteristics at various time points.

### Immune-related characteristics for the ICDI signature

Based on the TIMER algorithm^27^, CIBERSORT algorithm^28^, quantiseq algorithm^29^, MCPcounter algorithm^30^, xCell algorithm^31^, and EPIC algorithm, the study explored the relationship between the ICDI signature and immune cell infiltration. Based on ESTIMATE algorithm^32^, immunescore and stromalscore were calculated by analyzing the specific gene expression features of immune and stromal cells to predict the infiltration of non-tumor cells. The above algorithms were done through the “IOBR” package.

The study also evaluated the relationship between ICDI signature and biomarkers of immunotherapy. The “easier” package^33^ was used to calculate Cytotoxic activity (CYT)^34^, Roh immune score^35^, chemokine signature (chemokines)^36^, Davoli immune signature (Davoli_IS)^37^, extended immune signature (Ayers_expIS), T cell-inflamed gene expression profile (GEP)^38^, immune resistance program (RIR)^39^ and tertiary lymphoid structure signature (TLS)^39^. TIDE scores were obtained from the TIDE website (http://tide.dfci.harvard.edu/)^40^.

### Drug sensitivity analysis

Based on the TCGA gene expression profiles and GDSC cell line expression profiles^41^, the pRRophetic algorithm provided a ridge regression model to estimate drug IC50. The sensitivity of multiple medications to treat LUAD patients was assessed by employing the “pRRopheticPredict” package in the high ICDI score group and low ICDI score group.

### RNA extraction and quantitative real-time PCR (qRT-PCR)

With the help of the homo sapiens cell lines BEAS-2B, which were obtained from the Shanghai Cell Bank, Chinese Academy of Sciences, RT-qPCR was used to confirm the levels of RNA expression. The cell lines’ total RNA was extracted using the TRIzol (Invitrogen) reagent. Using the HiScript II Reverse Tran-scriptase (Vazyme) kit, the reverse transcription technique was carried out. Using the SYBR Green Supermix kit from Bio-Rad, the relative expression of RNA was determined by qPCR and normalized to the GAPDH expression level using the 2-ΔΔ (ct) technique. RNA-specific primers were synthesized in Wuhan GeneCreate Biological Engineering Co., Ltd.

### Statistical analysis

All data processing was conducted by R software (v.4.2.2). Statistical differences between groups were determined by Student’s t-test for normally distributed variables, and non-normally distributed variables, statistical differences between groups were determined by the Wilcoxon test.

## Results

### Genetic characteristics and transcriptional changes in ICD-related genes in LUAD

Summarized 34 ICD-related genes were identified through a large-scale meta-analysis^11^. The expression of 34 ICD genes in LUAD samples and normal samples was first analyzed (Figure S1A), and most of the ICD genes’ expressions were significantly different except for ATG5, IL10, CD8A, and CD8B. Secondly, the location of ICD-related genes in the human genome was analyzed (Figure S1B). the variation of ICD-related genes in LUAD patients in the TCGA cohort was also assessed. The results showed that approximately 69.63% (188/270) of LUAD patients had mutations in ICD-related genes, and the top 20 mutations in ICD-related genes were displayed in the study, with the highest frequency of mutations in TLR4 and NLRP3 (Figure S1C and Figure S1D).

The study also performed GO enrichment analysis of ICD-related genes (Figure S1E), which showed that, in terms of biological processes, the main enrichment was in various receptor activities. In terms of cellular components, the main enrichment was in the cytolytic granule and inflammasome complex. In terms of molecular functions, the main enrichment was in the biological processes of interleukin. In addition, KEGG enrichment analysis showed that ICD-related genes were enriched in the NOD-like receptor signaling pathway, Toll-like receptor signaling pathway, and Necroptosis. (Figure S1F).

### Construction and validation of the ICDI signature

**Figure 2:**
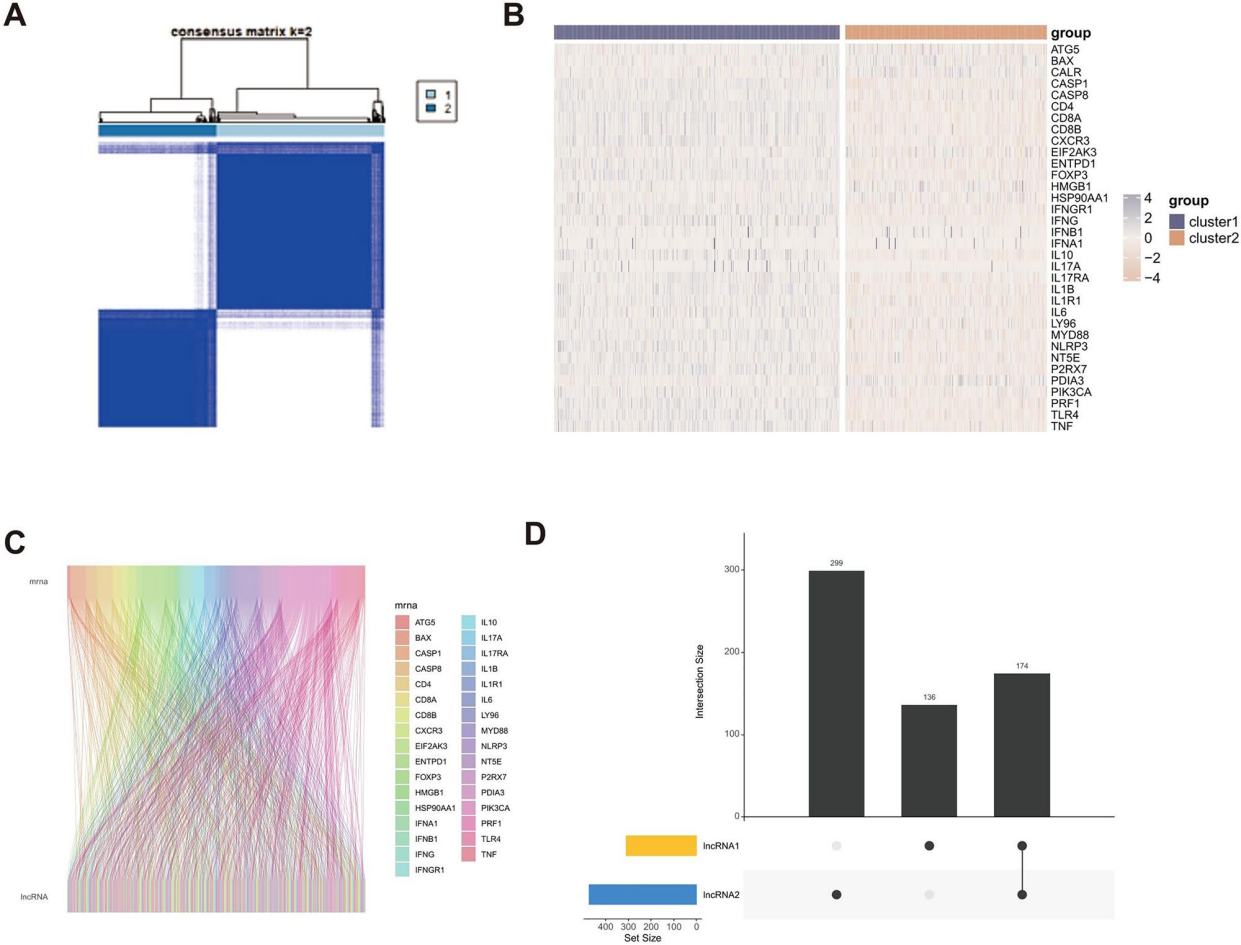
(**A**) Heatmap displaying 34 ICD gene expression profiles among normal and LUAD samples in the TCGA cohort. (**B**) The location of ICD-related genes in the human genome. (**C**) Single Nucleotide Polymorphism analysis of ICD-related genes in the TCGA cohort. (**E**) Bar plot displaying Gene Ontology analysis based on 34 ICD genes. (**F**) Bar plot displaying KEGG analysis based on 34 ICD genes.

A total of 1367 characteristic lncRNAs were selected by matching the training dataset with validation datasets for in-depth analysis. We employed consensus cluster analysis to partition the TCGA-LUAD dataset into two groups based on the high-expression and low-expression of ICD-related genes. Subsequently, 473 lncRNAs were identified by conducting differential expression analysis (Fig. 2A and B). These lncRNAs were then compared with the 300 lncRNAs obtained by Pearson correlation analysis (Fig. 2C) to identify 176 ICD-related lncRNAs (Fig. 2D). As a result, 24 ICD-related lncRNAs were ultimately identified by univariate Cox regression analysis (Supplementary Table 2).

**Figure 3:**
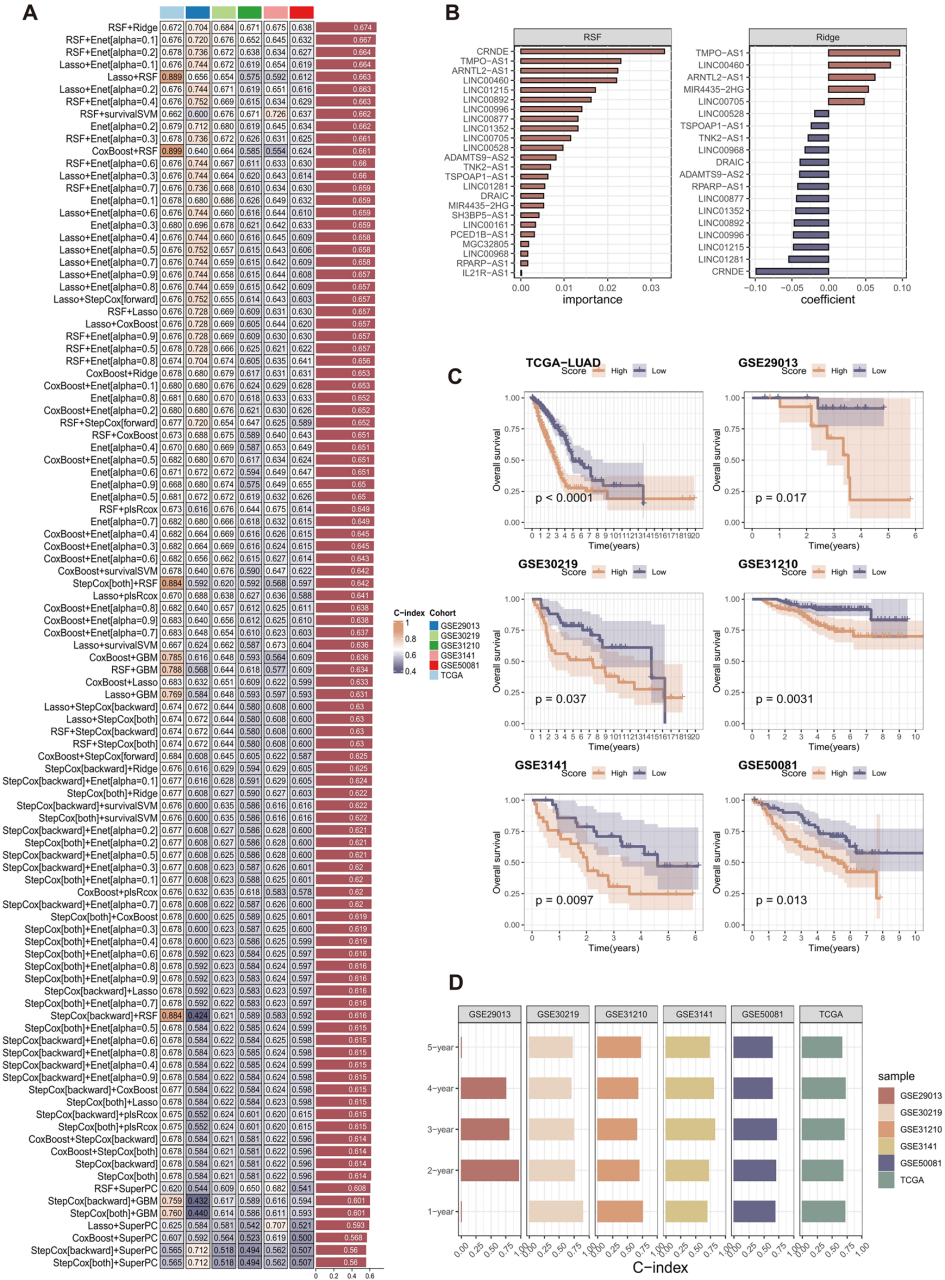
(**A**) A total of 101 combinations of machine learning algorithms for the ICDI signature via a tenfold cross-validation framework based on the TCGA-LUAD cohort. The C-index of each signature was calculated across validation datasets, including the GSE29013, GSE30219, GSE31210, GSE3141, and GSE50081 cohort. (**B**) 24 ICD-related lncRNAs’ importance ranking in the RSF algorithm and 19 lncRNAs enrolled in the ICDI signature coefficient finally obtained in the Ridge algorithm. (**C**) Kaplan–Meier survival curve of OS between patients with a high score of ICDI signature and with a low score of ICDI signature in TCGA-LUAD, GSE29013, GSE30219, GSE31210, GSE3141, and GSE50081 cohort. (**D**) Receiver operator characteristic (ROC) analysis for ICDI signature in TCGA-LUAD, GSE29013, GSE30219, GSE31210, GSE3141, and GSE50081 cohort.

A total of 24 ICD-related lncRNAs were inputted into a comprehensive machine-learning model, which encompassed the 10 aforementioned methodologies for creating prognostic signatures. Figure 3A illustrated the acquisition of a total of 101 prognostic models. The predictive signature created by the combination of RSF + Ridge had the greatest mean C index of 0.674, as determined by analyzing the training and test cohorts. This signature was identified as the ICDI signature, (Fig. 3A and B). The obtained equation is as follows (see Supplementary Table 3 for detail):

$${\text{ICDIscore}} = min \Vert \beta x - y \Vert_{2}^{2} + {\uplambda } \Vert \beta \Vert _{2}^{2}$$

As the elastic net mixing parameter, α was limited with 0 ≤ α ≤ 1. The λ is defined as $\uplambda =\frac{1-\alpha }{2}{\Vert \beta \Vert }_{2}^{2}+\alpha {\Vert \beta \Vert }_{1}$.

LUAD patients were categorized into two groups based on their ICDI score: a high-score group and a low-score group. The median value was used as the cut-off point. Consistent with expectations, LUAD patients with low ICDI scores exhibited higher overall survival rates in the TCGA-LUAD, GSE29013, GSE30129, GSE31210, GSE3141, and GSE50081 datasets (Fig. 3C).

The AUC values of 1-, 2-, 3-, 4-, and 5-year for the ICDI signature in the TCGA-LUAD cohort were estimated as 0.709, 0.678, 0.697, 0.716, and 0.660, respectively (Fig. 3D), demonstrating that ICDI signature has promising predictive value for LUAD patients. It was validated in the GSE30219 cohort (0.891, 0.758, 0.744, 0.700, and 0.716), GSE31210 cohort (0.750, 0.691, 0.653, 0.677 and 0.718), GSE3141 cohort (0.690, 0.716, 0.819, 0.801 and 0.729), GSE50081 cohort (0.685, 0.694, 0.712, 0.638, and 0.639), and GSE3141 cohort (0.639, 0.697, 0.794, 0.670, and 0.521) (Fig. 3D). As a result of insufficient survival data, the GSE29013 cohort only computes the AUC values for 2-, 3-, and 4-year periods. Still, it possesses strong predictive capability (Fig. 3D).

**Figure 4:**
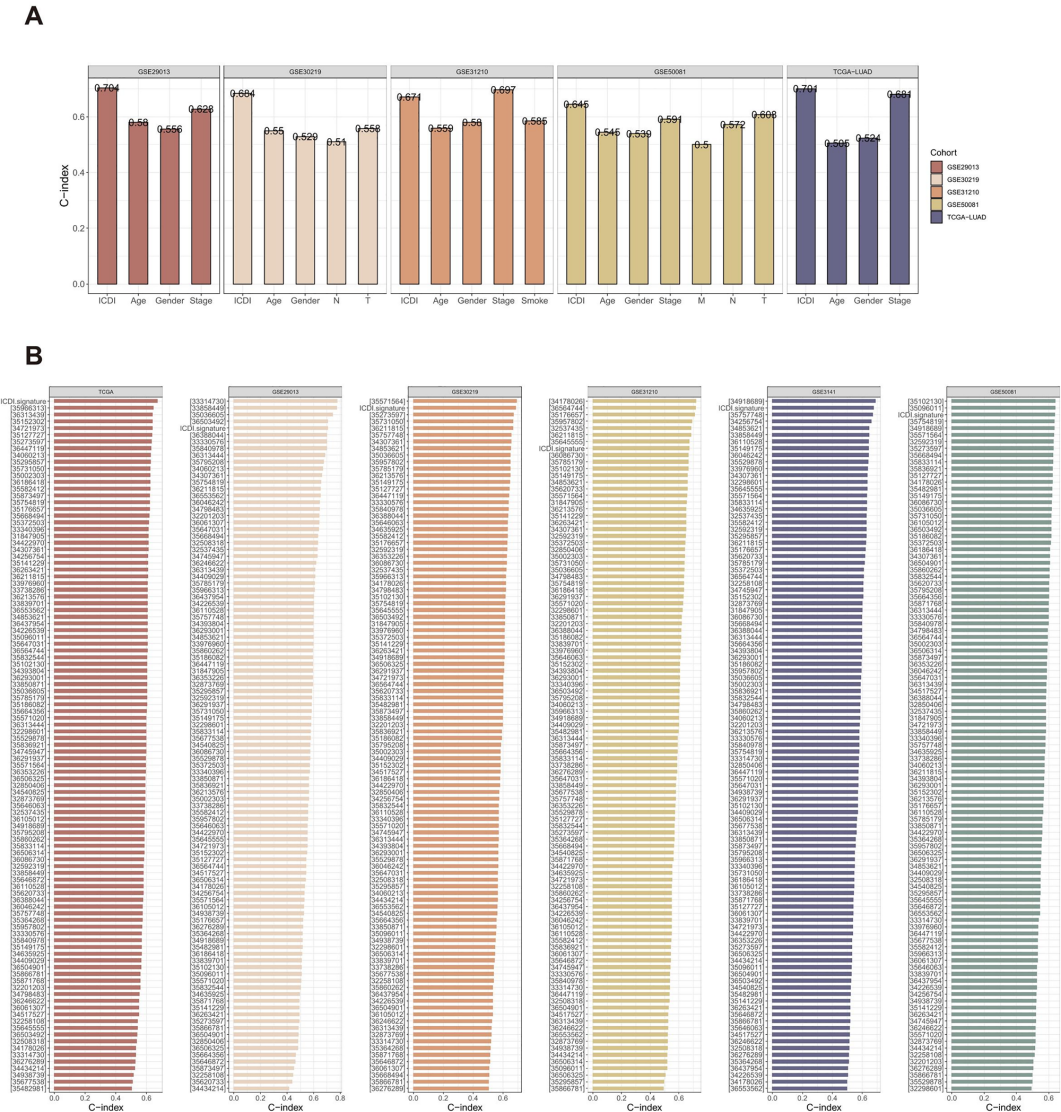
(**A**) The C-index of the ICDI signature and other clinical characteristics in the TCGA-LUAD, GSE29013, GSE30219, GSE31210, GSE3141 and GSE50081 cohorts. (**B**) The C-index of the ICDI signature and other signatures developed in the TCGA-LUAD, GSE29013, GSE30219, GSE31210, GSE3141 and GSE50081 cohorts.

In addition, we compared the predictive value of the ICDI signature with other clinical variables (Fig. 4A). The C-index of the ICDI signature was significantly higher than other clinical variables, covering staging, age, gender, etc.

### Comparison of prognostic signatures in LUAD

Gene expression analysis based on machine learning can be leveraged to predict the outcome of diseases, which in turn can facilitate in early screening of diseases, as well as in researching new therapeutic modalities. Substantial predictive signatures have emerged in recent years. To compare the ICDI signature with published signatures, we searched for LUAD-related disease prediction model articles. Excluding articles with unclear prediction model formulas and missing corresponding gene expression data in the training and validation groups, 102 LUAD-related predictive signatures were finally enrolled (Supplementary Table 4). These signatures contained various kinds of Biological processes, such as cuproptosis, ferroptosis, autophagy, epithelial-mesenchymal transition, acetylation, amino acid metabolism, anoikis, DNA repair, fatty acid metabolism, hypoxia, Inflammatory, N6-methyladenosine, mitochondrial homeostasis, and mTOR, which was established in TCGA-LUAD, GSE29013, GSE30219, GSE31210, GSE3141, and GSE50081 and compared with the C-index of ICDI, it can be seen that the ICDI signature outperformed the majority of signatures in each cohort (Fig. 4B).

### The ICDI signature’s potential as a biomarker for immunotherapy

**Figure 5:**
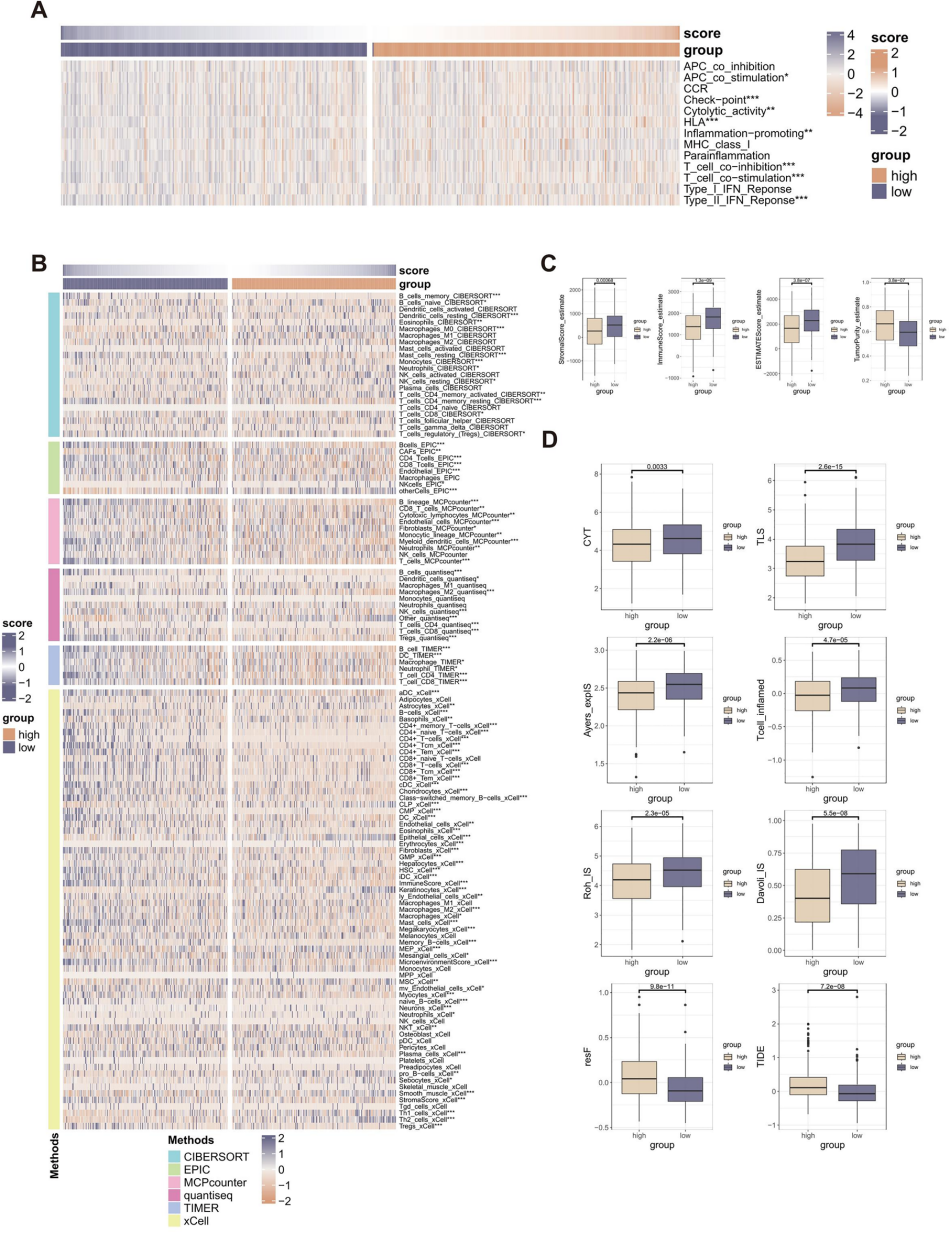
(**A**) Heatmap displaying the correlation between the ICDI signature and 13 immune-related processes. (**B**) Heatmap displaying the correlation between the ICDI signature and immune infiltrating cells. (**C**) Box plot displaying the correlation between the ICDI signature and The ESTIMATE Immune Score, ImmuneScore, StromalScore, and TumorPurity. (**D**) Box plot displaying the correlation between the ICDI signature and immune modulators.

To investigate the contribution of ICDI features in the LUAD TIME, we evaluated the correlation of ICDI features with immune infiltrating cells and immune-related processes. Based on TIMER algorithm, CIBERSORT algorithm, quantiseq algorithm, MCPcounter algorithm, xCell algorithm, and EPIC algorithm, the ICDI signature was correlated with most immune infiltrating cells except for a few (such as activated NK cells and CD8 + naive T cells) (Fig. 5A). Based on the ssGSEA algorithm, the ICDI signature was significantly correlated with most immune-related processes (Fig. 5B). Based on the ESTIMATE algorithm, the ICDI signature was negatively correlated with StromalScore, ImmuneScore, and ESTIMATEScore, and positively correlated with TumorPurity (Fig. 5C), as expected.

In addition, the study also evaluated the relationship between ICDI signature and known immune modulators (CYT, TLS, Davoli_IS, Roh_IS, Ayers_expIS, TIS, RIR, and TIDE) (Fig. 5D). The values of most of the immune modulators (CYT, TLS, Davoli_IS, Roh_IS, Ayers_expIS, and TIS) were significantly higher in the low ICDI signature scores group. The RIR values and TIDE score were all significantly higher in the high ICDI signature scores group, which suggested a higher potential for immunological escape (Fig. 5D) All of these displayed ICDI signature was a potential immunotherapeutic biomarker.

**Figure 6:**
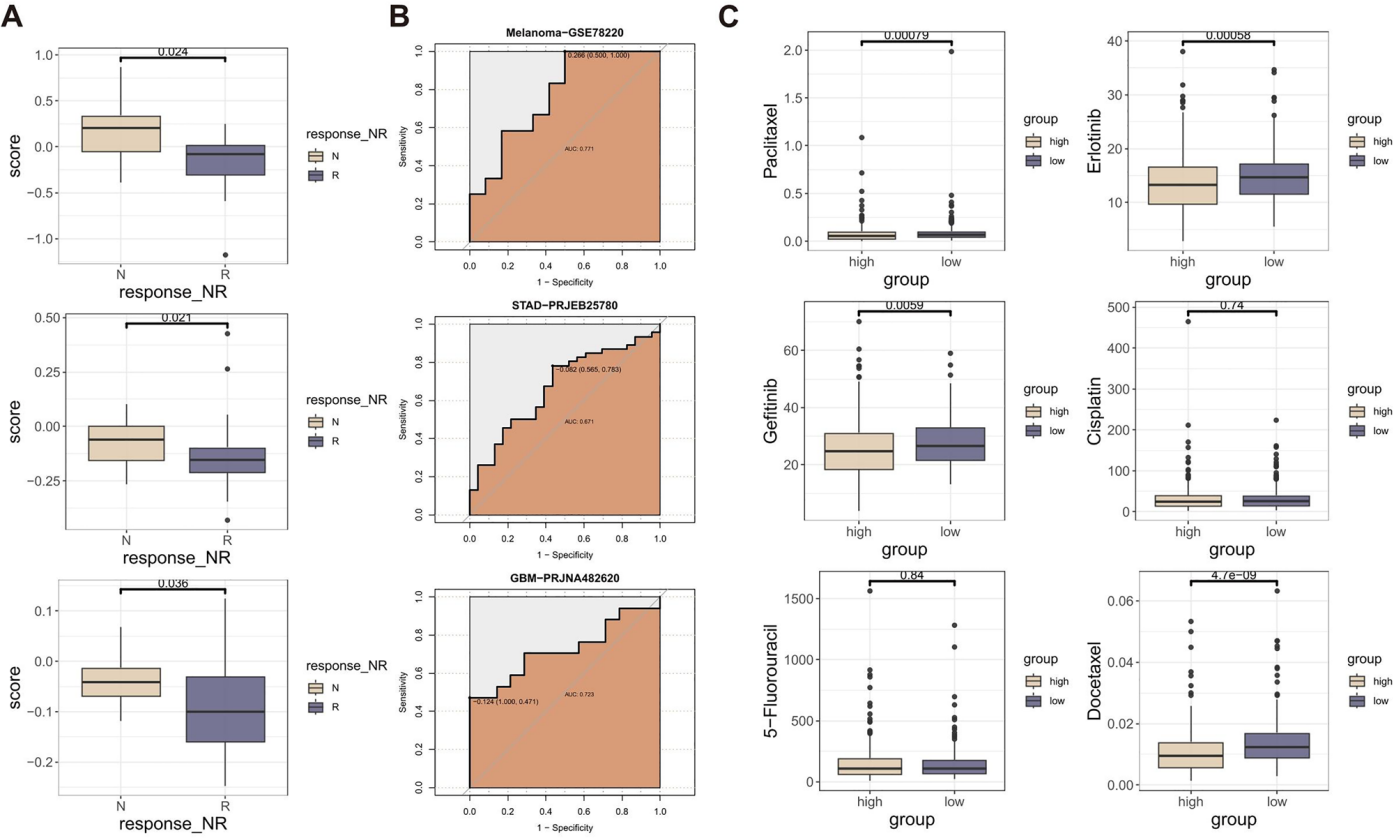
(**A**) Box plot displaying the correlation between the ICDI signature and immunotherapy response in the immunotherapy dataset (Melanoma-GSE78220, STAD-PRJEB25780, and GBM-PRJNA482620). (**B**) ROC curves of ICDI signature to predict the benefits of immunotherapy in the immunotherapy dataset (Melanoma-GSE78220, STAD-PRJEB25780, and GBM-PRJNA482620). (**C**) Box plot displaying the correlation between the ICDI signature and chemotherapy drugs.

To further investigate the potential of ICDI signature as an immunotherapeutic biomarker, the study calculated ICDI scores for each immunotherapy cohort respectively to appraise its predictive valuation. The findings indicated that those with a low ICDI score were more prone to derive advantages from immunotherapy. (Fig. 6A) The receiver operating characteristic (ROC) analysis conducted in the study showed that the ICDI signature exhibited a consistent ability to predict the efficacy of immunotherapy-based treatment. This finding was further supported by the analysis of immunotherapy datasets, including cohort Melanoma-GSE78220, STAD-PRJEB25780, and GBM-PRJNA482620, which yielded ROC values of 0.771, 0.671, and 0.723, respectively (Fig. 6B).

### Implications of ICDI signature for chemotherapy

Chemotherapy resistance is a significant barrier to the effectiveness of chemotherapy and targeted therapy in treating advanced lung cancer. We analyzed to determine the drug sensitivities of various chemotherapeutics in living organisms. We then compared the drug sensitivities using the ICDI signature. Individuals with low ICDI scores exhibited a notable rise in sensitivity to erlotinib, gefitinib, docetaxel, and paclitaxel. However, there was no significant variation in sensitivity to cisplatin and 5-fluorouracil. (Fig. 6C) The study offers instructions on the administration of chemotherapeutic medications in individuals with LUAD.

## Discussion

**Figure 7:**
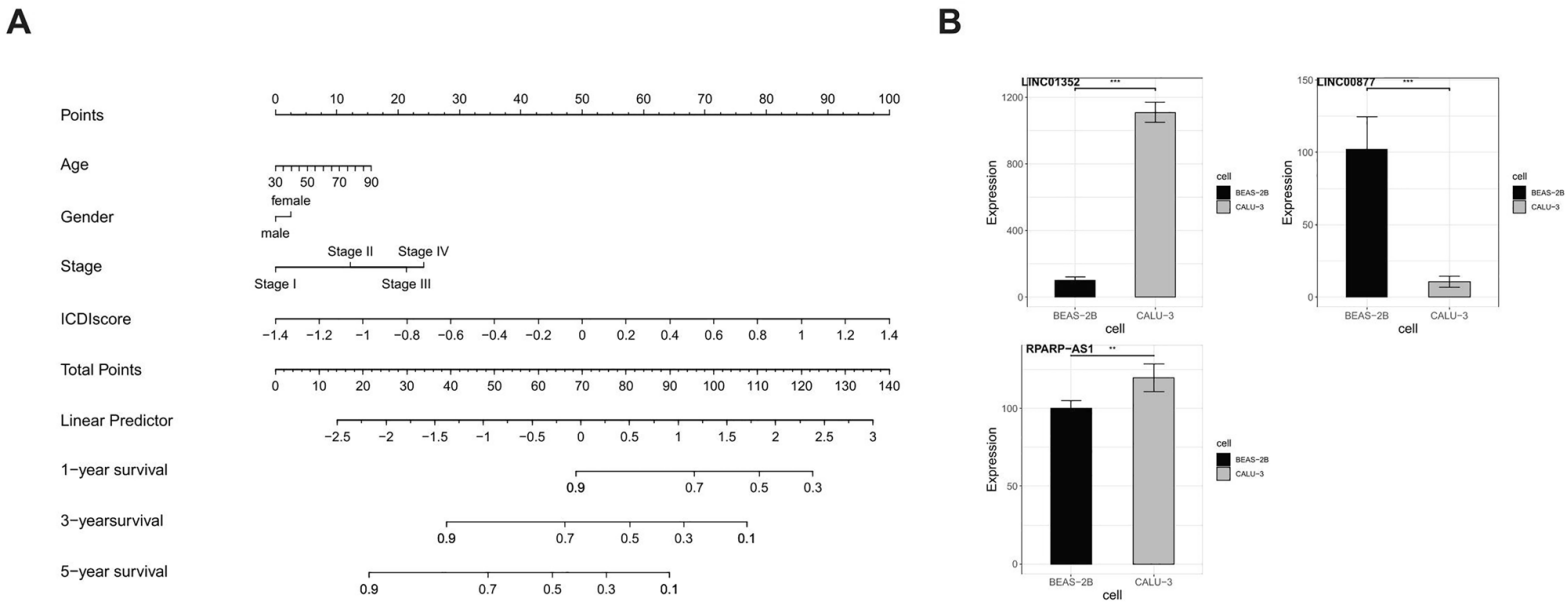
(**A**) Nomogram was established to predict the prognostic of LUAD patients. (**B**) Bar plot displaying the expression of LINC01352, LINC00877, and RPARP-AS1 in BEAS-2B and CALU-3 cells by RT-qPCR.

There are accumulated articles on constructing disease prognostic signatures based on machine learning algorithms now, of which most signatures are constructed only by lasso + StepCox algorithm combination, without robustness and stability. The study utilized Pearson correlation analysis, consensus cluster analysis, differential expression analysis, and univariate regression analysis to identify 24 ICD-related lncRNAs. A combination of methods ensured the dependability of identifying ICD-related lncRNAs. The most efficient ICDI signature was subsequently formed by combining the outcomes acquired from 101 machine learning methods. The assessment of ICDI signatures’ reliability involves the integration of TCGA and several GEO data sets, utilizing AUC and C-index metrics. The efficacy of this ICDI signature is assessed by comparing it to 102 previously released ICDI signatures. To facilitate computation, a nomogram was created, integrating the factors of age, gender, stage, and ICDI score (Fig. 7A). Furthermore, the immune characterization and drug sensitivity study provided evidence of the ICDI signature’s ability to effectively direct immunotherapy and chemotherapy.

Among the 19 ICD-related lncRNAs in our final screening for ICDI signature, most have been studied and preliminarily validated the function in cancer onset and progression.

ARNTL2-AS1 is associated with mRNA ARNTL2, which upregulates of ACOT7 promotes NSCLC cell proliferation through inhibition of apoptosis and ferroptosis^42^. Knockdown of TMPO-AS1 remarkably suppresses LUAD cell growth, induced apoptosis as well as G1/S arrest, and inhibited LUAD cell invasion, whereas overexpression of TMPO-AS1 exerts the opposite effects^43^. DRAIC/miR-3940-3p axis may be involved in the progression of LUAD and can be developed to promising prognostic factors^44^. LINC00996, significantly downregulated in LUAD, can inhibit cell proliferation, clonal formation, migration, and invasion in LUAD cell lines^45^. TNK2-AS1/microRNA-125a-5p axis promotes tumor growth and modulated phosphatidylinositol 3 kinase/AKT pathway^46^. ADAMTS9-AS2 plays an inhibitory role in LUAD cells^47^. TSPOAP1-AS1, downregulated in the LUAD group, plays a role in the prognosis of LUAD patients as cuproptosis-related lncRNA^48^. The aberrant upregulation of MIR4435-2HG in LUAD can upregulate related transcription factors and promote the EMT process^49^. LINC00892 has the potential as a biomarker of metastasis in T1 LUAD^50^. LINC00460 is upregulated in the LUAD group and played an important role in the control of oncogenes and tumor suppressors^51^. LINC01281 is downregulated in the LUAD group and therefore may be a prognostic factor for patients with LUAD^52^. LINC00968, upregulated in the LUAD group, is involved in the Wnt signaling pathway, which is involved in the growth, migration, and invasion of NSCLC^53^. LINC01215 inhibits the inflammatory response of tumors by increasing CD47 expression against M1 macrophages^54^. CRNDE in NSCLC cell lines with significantly upregulated expression promoted the proliferation and growth of NSCLC cells through activation of PI3K/AKT signaling^55,56^.

RT-qPCR verified the expression of the other lncRNAs (LINC01352, LINC00877, and RPARP-AS1) (Supplementary Table 5) in BEAS-2B and CALU-3 cells (Fig. 7B). The decreased expression of LINC00877 was observed in CALU-3 LUAD cells compared to BEAS-2B normal cells. Meanwhile, the increased expression of LINC01352, and RPARP-AS1 was observed in CALU-3 LUAD cells compared to BEAS-2B normal cells. All of the indicated results showed statistical significance and were also concordant with the results in the TCGA database. Overall, the accuracy of our risk profile was validated by the experimental results.

The study possesses distinctive characteristics when compared to the design of other prognostic models. Initially, a total of 24 ICD-related lncRNAs were identified using Pearson correlation analysis, consensus clustering, differential expression analysis, and single-factor regression analysis. This guarantees the dependability of lncRNAs associated with ICD. This work discovers the optimal ICDI signature by integrating 101 machine learning methods. The reliability of the ICDI signature was assessed by integrating data from both the TCGA and various GEO data sets. The reliability and superiority of this ICDI signature were assessed by employing various evaluation measures, such as AUC and C-index, and comparing it with 102 previously published signatures. Several immune infiltration algorithms, such as ssGSEA, TIMER, quanTIseq, MCP-counter, xCell, EPIC, and ESTIMATE, along with immunotherapy markers and datasets, assessed ICDI as a reference for guiding immunotherapy and chemotherapy.

Undoubtedly, the study possesses numerous deficiencies. Initially, we exclusively utilized datasets that included comprehensive lncRNA expression data from the ICDI signature to validate the model’s prognostic and immunotherapy guiding capabilities, as most datasets lacked complete lncRNA expression data. Subsequent data will be required to confirm the universality of the model and the efficacy of the immunotherapy guidance function. Furthermore, the verification of particular lncRNAs (LINC01352, LINC00877, and RPARP-AS1) in both normal and lung cancer cell lines was exclusively conducted using RT-qPCR. Additional research is required to determine the involvement of ICD-related lncRNAs in immunogenic cell death.

## Conclusion

In conclusion, the ICDI signature proposed in this study is a potential prognostic predictor for LUAD patients in overall survival and immunotherapy response.

### Supplementary Information

Supplementary Figure S1.

Supplementary Table.

## Data Availability

The analyzed data could be obtained from the TGCA database (https://portal.gdc.cancer.gov/), GEO database (http://www.ncbi.nlm.nih.gov/geo/), and TIDE database (http://tide.dfci.harvard.edu/). The code applied in the study is available from the corresponding author upon reasonable request.
