# Supplementary material for: Machine learning-based integration develops an immunogenic cell death-derived lncRNA signature for predicting prognosis and immunotherapy response in lung adenocarcinoma
Source: Sci Rep. 2024 May 22;14:11724. doi: 10.1038/s41598-024-62569-z (PMC11111459; doi:10.1038/s41598-024-62569-z)
Supplement: Supplementary file 1 — Supplementary Figure S1. [file 41598_2024_62569_MOESM1_ESM.docx]

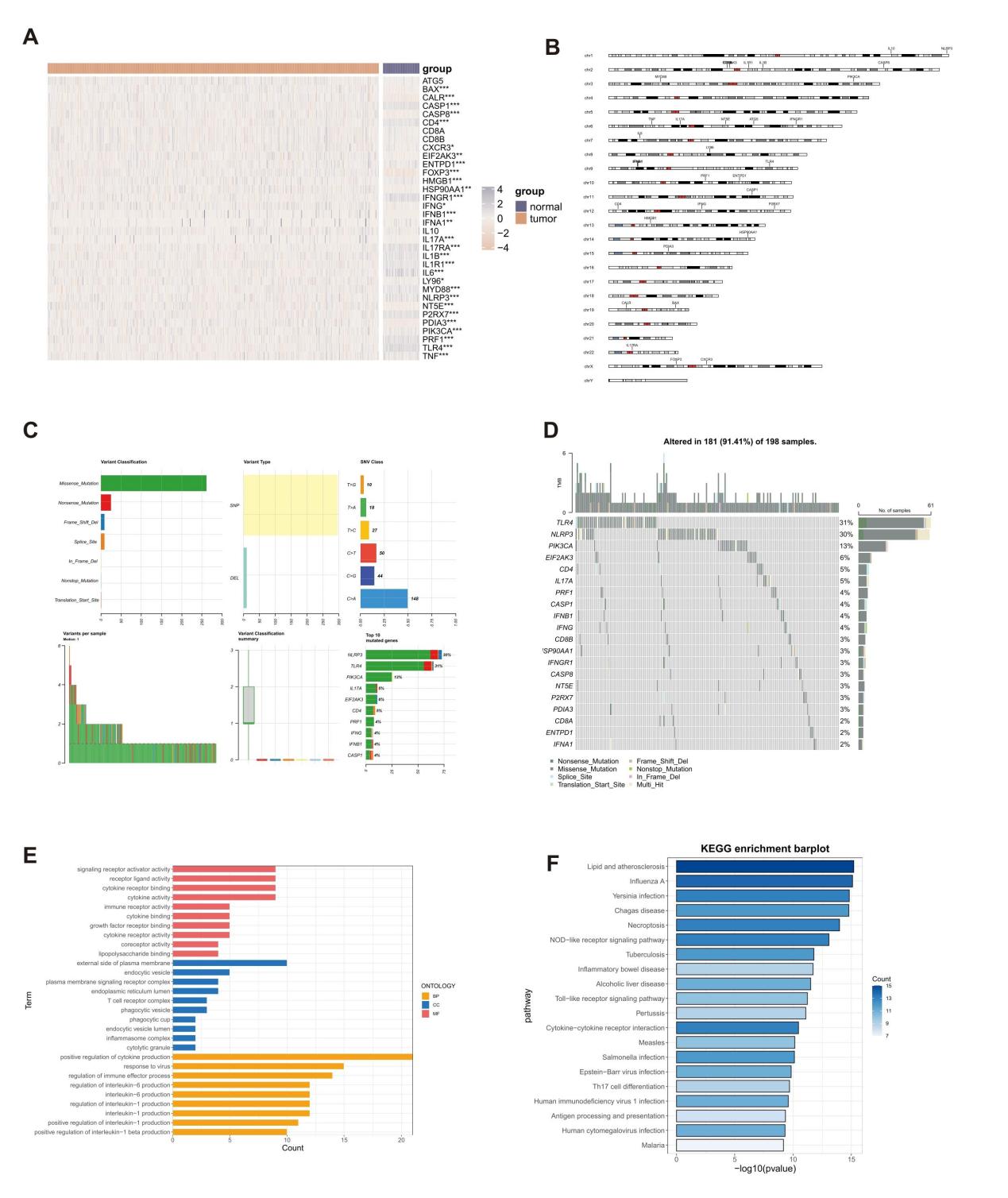


Figure. S1 (**A**) Heatmap displaying 34 ICD gene expression profiles among normal and LUAD samples in the TCGA cohort. (**B**) The location of ICD-related genes in the human genome. (**C**) Single Nucleotide Polymorphism analysis of ICD-related genes in the TCGA cohort. (**D**) Waterfall charts displaying the frequency of gene mutations. (**E**) Bar plot displaying Gene Ontology analysis based on 34 ICD genes. (**F**) Bar plot displaying KEGG analysis based on 34 ICD genes.
